# Endotracheal Surfactant and Budesonide Combination Therapy in Neonatal Acute Respiratory Distress Syndrome due to Late-Onset Sepsis

**DOI:** 10.34172/aim.31725

**Published:** 2024-10-01

**Authors:** Asli Okbay Gunes, Aydin Bozkaya

**Affiliations:** ^1^Neonatal Intensive Care Unit, Sanliurfa Training and Research Hospital, Sanliurfa, Turkey

**Keywords:** Acute respiratory distress syndrome, Budesonide, Endotracheal therapy, Neonates, Surfactant

## Abstract

**Background:**

Neonatal acute respiratory distress syndrome (NARDS) is an important cause of hypoxemic respiratory failure. This study aimed to investigate the short-term effects of endotracheal surfactant and budesonide combination therapy on NARDS secondary to late-onset neonatal sepsis (LONS).

**Methods:**

This was a retrospective, cross-sectional, and observational study. Newborns with NARDS due to LONS who received endotracheal surfactant and budesonide combination therapy between August 2022 and September 2023 were included in this study. Oxygenation status before endotracheal surfactant and budesonide treatment were compared with the values obtained two hours after treatment.

**Results:**

Among 20 neonates, 10 (50%) were diagnosed with severe NARDS, and 10 (50%) were diagnosed with moderate NARDS. The mean corrected gestational age was 33.3±2.9 w when endotracheal surfactant and budesonide were administered to the neonates. The need for the fraction of inspired oxygen (0.75 [0.57-1.00]% vs. 0.55 [0.44-0.80]%; mean difference [MD]: 17.50%, 95% confidence interval [CI]: 14.99 to 22.50) and oxygen saturation index (OSI; 8.03 [4.98-13.94] vs. 4.71 [4.11-8.93]; MD: 2.23, 95% CI: 1.22 to 3.24) decreased (*P*=0.001 and *P*<0.001, respectively) after endotracheal surfactant and budesonide treatment. However, preductal oxygen saturation (SpO_2_; 93 [91-94]% vs. 95 [94-96]%; MD: -3.50%, 95% CI: -5.00 to -2.00) increased significantly after endotracheal surfactant and budesonide treatment when compared to pre-treatment values (*P*<0.001).

**Conclusion:**

The reduction in oxygen demand and OSI, along with an increase in SpO_2_ after treatment compared to pre-treatment values, suggests that endotracheal surfactant and budesonide combination therapy could be an effective option to improve oxygenation in NARDS secondary to LONS.

## Introduction

 Neonatal acute respiratory distress syndrome (NARDS) is a serious cause of severe hypoxemic respiratory failure characterized by surfactant catabolism and widespread lung inflammation, occurring approximately in 1.5% of neonatal intensive care unit (NICU) admissions with an overall mortality rate of 17%-24%.^1,2^ The Montreux definition of NARDS was published in 2017 and has been used ever since, especially in scientific research.^1^ Since that time, no specific treatment has been found for NARDS, and the treatment is managed according to the underlying cause. In addition, the neonates are provided with general support such as adequate ventilation, nutrition, fluid, and electrolyte therapy, as well as therapeutic support, including antibiotics, inhaled nitric oxide, inotropes, and the like.^1-3^ It was attempted to develop a predictive model for early diagnosis of NARDS based on the Montreux definition, and meconium-stained amniotic fluid, higher absolute neutrophil count, lower platelet count, and lower serum calcium level emerged as the independent risk factors for NARDS.^4^

 Although exogenous surfactant therapy is not recommended as a routine treatment in either NARDS or pediatric ARDS, it is an effective and well-tolerated treatment in selected cases.^2,5-7^ On the other hand, budesonide is a candidate for effective treatment of NARDS due to its potential anti-inflammatory effect.^8-13^ Budesonide belongs to the corticosteroid drug class and exerts anti-inflammatory effects by inhibiting the production of inflammatory genes, reducing cytokine formation, inhibiting the activation of eosinophils, and suppressing the activation of inflammatory cells.^14^ In this respect, budesonide has long been used to prevent bronchopulmonary dysplasia (BPD) in premature newborns with respiratory distress syndrome (RDS) by inhalation or intratracheal administration and seems to be effective in treatment.^15,16^ When compared to endotracheal surfactant alone, endotracheal surfactant and budesonide treatment in premature infants with RDS has been found to have beneficial effects on short-term respiratory outcomes such as decreased severity of BPD and decreased need for mechanical ventilation, without any short-term morbidity, and apparent long-term adverse effects on physical growth, neuromotor, and cognitive functions.^9,12,17-19^ However, the effect of endotracheal surfactant and budesonide combination therapy in preterms on BPD alone or the combined outcome of death or BPD is still under investigation.^8,9,12,13,20-24^ Additionally, the benefit of budesonide in the transient tachypnea of ​​the newborn and meconium aspiration syndrome has been investigated, but no clear conclusion has been reached regarding its effectiveness in treatment.^25,26^

 Neonatal ARDS can be classified into primary/direct or secondary/indirect, infectious or noninfectious, and perinatal (≤ 72 hours after birth) or late-onset ( > 72 hours after birth) groups, according to the underlying cause and time of occurrence.^2^ Sepsis is the most common cause of NARDS and leads to an indirect and infectious NARDS.^2^ Premature birth, cesarean delivery, early-onset neonatal sepsis, and septic shock have been found to be independently associated with sepsis-related NARDS in near-term and term newborns when compared to sepsis plus the non-NARDS group.^27^ NARDS is related to a higher need for respiratory support and better responsiveness to surfactant therapy when compared to pediatric ARDS.^28^ Surfactant has been confirmed to improve oxygenation in NARDS caused by pneumonia at gestational age beyond 34 weeks without reductions in mortality, ventilator or oxygen time, or major morbidity.^29^ However, the need for two or more doses of surfactant was found to be associated with increased mortality compared to one or zero doses of surfactant in NARDS.^30^ Deliloglu et al^31^ reported two cases of NARDS due to late-onset neonatal sepsis (LONS) that were successfully treated with a combination of endotracheal surfactant and budesonide. In that case report, following endotracheal surfactant and budesonide combination therapy given the lack of improvement in oxygenation indexes (OI) despite optimal ventilation and surfactant treatment, a stepwise decrease in OI was observed, and both patients were discharged home with spontaneous breathing of room air.^31^ In this retrospective, cross-sectional, and observational study, it was aimed to evaluate the short-term effects of endotracheal surfactant and budesonide combination therapy in NARDS secondary to LONS based on the current literature.

## Materials and Methods

 This was a retrospective cross-sectional and observational study. Newborns who were diagnosed with NARDS secondary to LONS and received endotracheal surfactant and budesonide combination therapy between August 2022 and September 2023 were included in the study. Ethical approval for the study was obtained from the University Clinical Research Ethics Committee (date: 18.03.2024, number: 24.02.72). The study was completed in accordance with the Declaration of Helsinki as revised in 2013. Written informed consent was obtained from both the mothers and fathers of all the patients before applying endotracheal surfactant and budesonide treatment, and consent forms were kept in patients’ files. Neonates who had major congenital anomalies incompatible with life or who died within two hours after endotracheal surfactant and budesonide treatment were excluded from the study. Twenty-two neonates were initially eligible for enrollment. Two of these neonates were excluded from the study because one was diagnosed with fatal skeletal dysplasia during follow-up, and one died two hours after endotracheal surfactant and budesonide treatment. Thus, a total of 20 newborns underwent analysis.

###  Sepsis Evaluation Protocol

 We used our national guideline on neonatal infections’ diagnosis and treatment for sepsis evaluation in our NICU.^32^ In the presence of any clinical signs of sepsis according to the European Medicines Agency sepsis scoring system, a full sepsis evaluation was performed, including complete blood count, C-reactive protein, procalcitonin, hepatic and renal function tests, blood, cerebrospinal fluid, and urine cultures. The other investigated parameters were lung radiography if there was a respiratory problem and tracheal aspirate culture for mechanically ventilated neonates in the presence of suspected pneumonia. Suspected sepsis was considered if the laboratory findings were consistent with sepsis, but the blood culture was negative, and confirmed sepsis was considered in the presence of a positive blood culture result in addition to sepsis findings.^32^ Considering the etiologic agents of sepsis in our clinic, newborns with suspected LONS were treated with vancomycin for Gram-positive agents and amikacin or meropenem for Gram-negative agents until the culture results were obtained.

###  Ventilation Strategy

 Respiratory care was provided in accordance with the formal NICU protocol, and lung protective ventilation strategies were adopted. Respiratory failure was defined as the presence of the ongoing signs of respiratory distress and associated acidosis (pH < 7.25), hypercarbia (partial pressure of arterial carbon dioxide [PCO_2_] > 60 to 65 mm Hg), and hypoxemia (fraction of inspired oxygen [FIO_2_] > 40%). Intubation was reserved for neonates with respiratory failure and severe apnea on maximal nasal intermittent positive pressure ventilation (positive end-expiratory pressure ≥ 8-10 cm H_2_O and positive inspiratory pressure ≥ 20-25 cm H_2_O) support.^33^ Assist-control ventilation mode with volume guarantee and synchronized intermittent mandatory ventilation with volume guarantee were preferred as conventional mechanical ventilation (CMV) modes in intubated infants. Target tidal volumes for volume guarantee were set at 4–8 mL/kg. High-frequency oscillatory ventilation (HFOV) was used as a rescue mode ventilation and was utilized for neonates with respiratory failure on CMV (mean alveolar pressure [MAP] > 8 cm H_2_O and FIO_2_ > 40%) support.

###  Neonatal Acute Respiratory Distress Syndrome Definition

 Newborns who were diagnosed with LONS and were experiencing respiratory failure were evaluated for the presence of NARDS according to the Montreux definition of NARDS. Newborns meeting the Montreux criteria were candidates for the study, including (1) acute onset within one week after a known or suspected clinical insult and (2) diffuse, bilateral, irregular opacities or infiltrates, or complete opacification of both lungs on the chest X-ray that cannot be fully explained by local effusions, atelectasis, RDS, transient tachypnea of the newborn, or congenital anomalies. The other criteria were (3) the echocardiographic confirmation of the absence of congenital heart disease explaining the pulmonary edema and (4) the severity of NARDS categorized based on the OI as mild (4 ≤ OI < 8), moderate (8 ≤ OI < 16), and severe (OI ≥ 16).^1^ The first Montreux criterion was met with the need for HFOV ventilation developing in newborns ventilated in another mode or the need for a greater than 20% increase in MAP in newborns ventilated in the HFOV mode after the diagnosis of LONS. The oxygen saturation index (OSI) was used instead of OI, as we did not routinely perform arterial blood draws for testing blood gases. The OSI was calculated as MAP × FIO_2_ × 100 / preductal oxygen saturation (SpO_2_) as measured by pulse oximetry and was recorded two hours before and after endotracheal surfactant and budesonide combination therapy. The OSI is known to be strongly predictive of clinically relevant OI (OI = 2xOSI) and a non-invasive method to assess the oxygenation status of neonates continuously.^34^

###  Intervention

 All patients underwent lung recruitment maneuvers to ensure optimal ventilation before endotracheal surfactant and budesonide treatment. However, patients who continued to have respiratory failure were administered poractant alfa (Curosurf^®^, Chiesi Pharmaceuticals, Parma, Italy) at a dose of 200 mg/kg and budesonide (Pulmicort^®^ nebulizing suspension, Astra Zeneca, London, UK) at a dose of 0.25 mg/kg endotracheally after the diagnosis of NARDS due to LONS confirmation. This formulation of poractant alfa (200 mg/kg) and budesonide (0.25 mg/kg) was speculated to be potentially appropriate in terms of safety and efficacy to be used in premature for optimal surfactant function and budesonide stability.^35^ Surfactant and budesonide were mixed in the same syringe in a sterile fashion and applied to all patients by the same two investigators. To standardize data collection and ensure consistency, the investigators applied endotracheal surfactant and budesonide combination therapy using the same procedure as described in the literature.^31^ When budesonide is supplemented, both the surface tension-reducing properties and chemical stability of poractant alfa are known to be maintained for at least 24-48 hours.^36^

 Partial pressure of carbon dioxide (PCO_2_), pH, bicarbonate, base excess, lactate, FIO_2_, MAP, SpO_2_, OSI, and blood pressure (BP) were recorded before and two hours after endotracheal surfactant and budesonide combination therapy. BP was measured noninvasively with appropriate cuffs. A chest X-ray was taken to diagnose NARDS in all patients. In addition, a repeat chest X-ray was taken 4-6 hours after endotracheal surfactant and budesonide treatment was started. Extracorporeal membrane oxygenation could not be performed in our city.

###  Outcome Measurements

 The primary outcome of the study was to compare blood gas values, oxygenation status, BP, and chest X-ray findings of the neonates before and after endotracheal surfactant and budesonide treatment. The secondary outcome of the study was to determine the rates of BPD and mortality and the length of hospital stay. The demographic and clinical findings, laboratory and culture results, length of hospital stay and mortality were extracted from the patients’ files. The newborns’ resting BP was measured non-invasively using appropriate cuffs with automatic devices. The blood gas device that belonged to our NICU was used in the study. The devices for measuring BP or blood gases were calibrated at each measurement and when deemed necessary, and also all devices were periodically calibrated by our hospital’s quality unit.

 Potential confounding factors, such as the small sample size, the absence of a control group, and the lack of long-term follow-up, were mitigated by using the Montreux definition for NARDS, ensuring the homogeneity of NARDS etiology, and administering the same dose of endotracheal surfactant and budesonide with a standardized procedure.

###  Statistical Analyses

 The Statistical Package for Social Sciences (version 25.0, IBM, Armonk, NY, ABD) was utilized for statistical analyses. A previous study reported that surfactant treatment decreased OI from 11 to 7.^29^ Based on this data, it was also hypothesized that endotracheal surfactant and budesonide therapy was expected to reduce OI from 11 to 7, indicating a reduction of OSI from 5.5 to 3.5, and the standard deviation of the OI was accepted as 6. The detection of such a difference required 20 patients using a power of 80% and a two-sided alpha value of 5%. Categorical variables were expressed as percentages and frequencies. The continuous variables were distributed non-normally, and therefore they were presented as median values (interquartile range [IQR], p25-p75). Mean differences (MDs) with 95% confidence intervals (CIs) and effect sizes were calculated. Considering that the values recorded before and after the endotracheal surfactant and budesonide combination treatment were non-normally distributed, the Wilcoxon test was used to compare the values before and after the treatment, and a *P*-value < 0.05 was considered to be statistically significant.

## Results

 During the study period, 28 618 births occurred in our hospital, and 2768 newborn patients were admitted to our NICU. The frequency of NARDS due to LONS in babies admitted to the NICU was 0.8%. Twenty-two neonates received endotracheal surfactant and budesonide treatment for NARDS due to LONS throughout the study period. Two neonates were excluded due to the exclusion criteria mentioned above, and a total of 20 neonates underwent analysis. The mean gestational age and the mean corrected gestational age were found to be 28.75 ± 16.5 weeks and 33.3 ± 2.9 weeks, respectively, when endotracheal surfactant and budesonide were administered. The median birth weight and the median weight were found to be 1120 g (770-2100) and 1345 g (700-3120), respectively, when endotracheal surfactant and budesonide were administered. All patients included in the study had to be ventilated in the HFOV mode due to severe respiratory failure. There was growth in blood culture in 14 (70%) patients. Moreover, 16 (80%) patients were diagnosed with BPD, and 6 (30%) patients died. Four (20%) patients developed pulmonary hypertension and were given inhaled nitric oxide treatment, and patients with pulmonary hypertension died. The demographic and clinical characteristics, laboratory results are summarized in Table 1.

**Table 1:** Demographic and Clinical Characteristics, and Laboratory Results (N = 20)

**Characteristics**	**Values**
Demographic characteristics	
Female^a^	7 (35)
Cesarean section^a^	14 (70)
1st minute APGAR score^b^	5.2 ± 1.39
5th minute APGAR score^b^	7.3 ± 0.86
Gestational age, w ^b^	29.2 ± 2.9
Postnatal age during treatment, day ^b^	28.75 ± 16.5
Corrected gestational age during treatment, w ^b^	33.3 ± 2.9
Birth weight, g ^c^	1120 (770-2100)
Weigth during treatment, g ^c^	1345 (700-3120)
Clinical characteristics	
Day of antibiotics during treatment ^b^	6.65 ± 3.77
Growth in blood culture, yes ^a^	14 (70)
Broncopulmonary dysplasia ^a^	16 (80)
Length of hospital stay ^b^	55.9 ± 23.1
Mortality ^a^	6 (30)
Laboratory results during sepsis	
Hemoglobin, g/dL ^b^	12.75 ± 2.53
White blood cell, /μL ^b^	17.395 ± 6.480
Thrombocyte, /μL ^b^	145.750 ± 105.490
C-reactive protein, mg/L ^c^	25.5 (1.8-120)
Procalcitonin, µg/l ^c^	1.75 (0.15-10.35)

^a^ Number (percentage). ^b^ Mean ± standard deviation. ^c^ Median (minimum-maximum).

 According to OI calculated as 2xOSI, 10 (50%) patients were diagnosed with severe NARDS, 10 (50%) were diagnosed with moderate NARDS, and the mortality rate was 30% in both the severe and moderate NARDS groups. It was found that PCO_2_ (62.70 [56.13–66.00] mm Hg vs. 53.50 [44.85–63.00] mm Hg; MD: 9.30 mm Hg, 95% CI: 4.00 to 13.95), FIO_2_ (0.75 [0.57–1.00]% vs. 0.55 [0.44–0.80]%; MD: 17.50%, 95% CI: 14.99 to 22.50), and OSI (8.03 [4.98–13.94] vs. 4.71 [4.11–8.93]; MD: 2.23, 95% CI: 1.22 to 3.24) decreased significantly after endotracheal surfactant and budesonide treatment when compared to pre-treatment values (*P* = 0.001, *P* < 0.001, and *P* < 0.001, respectively). Conversely, pH (7.20 [7.09v7.28] vs. 7.22 [7.12–7.30; MD: -0.03; 95% CI: -0.08 to -0.02) and SpO_2_ (93 [91-94]% vs. 95 [94–96]%; MD: -3.50%, 95% CI: -5.00 to -2.00) increased significantly after endotracheal surfactant and budesonide treatment when compared to pre-treatment values (*P*= 0.007 and *P* < 0.001, respectively). No difference was found when comparing bicarbonate, base excess, lactate, and MAP values ​​recorded before endotracheal surfactant and budesonide treatment with those obtained after surfactant and budesonide treatment. After endotracheal surfactant and budesonide combination therapy, systolic (56 [47–66] vs. 61 [53–67] mm Hg; MD: -4.49 mm Hg, 95% CI: -7.00 to -1.50) and diastolic BP (32 [24–39] vs. 46 [38–50] mm Hg; MD: -4.00 mm Hg, 95% CI: -7.00 to -1.00) values were observed to have increased significantly compared to pre-treatment values (*P* = 0.016 and *P* = 0.017, respectively). However, mean arterial BP values were similar before and after treatment. Table 2 presents changes in laboratory results and clinical findings before and after endotracheal surfactant and budesonide treatment.

**Table 2:** Comparison of Laboratory and Clinical Findings Before and After Endotracheal Surfactant and Budesonide Treatment (N = 20)

**Variables**	**Before**^a^	**After**^a^	**Mean Difference (95% CI)**	**Effect Size**	***P*** **Value**^b^
pH	7.20 (7.09-7.28)	7.22 (7.12-7.30)	-0.03 (-0.08, -0.02)	-0.711	0.007
PCO_2_ (mm Hg)	62.70 (56.13-66.00)	53.50 (44.85-63.00)	9.30 (4.00, 13.95)	0.838	0.001
Bicarbonate (mmol/L)	20.65 (18.52-24.97)	21.10 (18-23.82)	0.60 (-1.55, 3.20)	0.150	0.620
Base excess (mmol/L)	-3 ([-4.78]- [-0.47])	-2.5 ([-4.35]- [-0.62])	-0.85 (-2.30, 0.95)	-0.271	0.295
Lactate (mmol/L)	2.79 (1.56-3.43)	2.29 (1.53-2.86)	0.20 (-0.39, -0.75)	0.181	0.490
FIO_2_	0.75 (0.57-1.00)	0.55 (0.44-0.80)	17.50 (14.99, 22.50)	1.000	0.001
Mean alveolar pressure (cmH_2_O)	10 (8.4-12.5)	9 (8-11.3)	1.00 (-1.47, 5.00)	0.567	0.053
SpO_2_ (%)	93 (91-94)	95 (94-96)	-3.50 (-5.00, -2.00)	-0.905	< 0.001
Oxygen saturation index	8.03 (4.98-13.94)	4.71 (4.11-8.93)	2.23 (1.22, 3.24)	0.838	< 0.001
Sistolic blood pressure (mm Hg)	56 (47-66)	61 (53-67)	-4.49 (-7.00, -1.50)	-0.632	0.016
Diastolic blood pressure (mm Hg)	32 (24-39)	46 (38-50)	-4.00 (-7.00, -1.00)	-0.626	0.017
Mean arterial blood pressure (mm Hg)	43 (34-48)	46 (39-50)	-3.50 (-6.00, 0.99)	-0.386	0.134

*Note*. CI: Confidence interval; FIO_2_: Fraction of inspired oxygen; PCO_2_: Partial pressure of carbon dioxide; SpO_2_: Preductal oxygen saturation. ^a^Median (interquartile range: p25-p75), ^b^Wilcoxon test.

## Discussion

 This study was conducted to determine the short-term effect of endotracheal surfactant and budesonide combination therapy for NARDS due to LONS. Based on the findings, PCO_2_, the need for FIO_2_, and OSI were lower, while pH and SpO_2_ were higher after treatment compared to pre-treatment values, while there was no difference in MAP before and after endotracheal surfactant and budesonide treatment. Detection of lower carbon dioxide levels and better oxygenation status ​​with similar ventilation mode and MAP suggested that endotracheal surfactant and budesonide therapy might be a ventilation-improving modality in NARDS due to LONS. On the other hand, systolic and diastolic BP increased significantly after endotracheal surfactant and budesonide treatment when compared to pre-treatment values, and this finding alarmed us that the treatment could lead to undesirable side effects by increasing BP. However, both systolic and diastolic BP values were in the normal range for corrected gestational age,^37^ and mean arterial BP values were similar before and after treatment, implying that there was no clinically significant increase in BP due to steroid instillation. This finding supports the view that the tracheal instillation of steroids potentially reduces systemic side effects with a lower circulating level compared to systemic use.^38^

 In the field of neonatology, there is always hesitancy in deciding on steroid therapy by any route, including systemic, inhaled, and endotracheal routes, due to fear of short- and long-term adverse consequences, especially unfavorable neurodevelopmental outcomes.^39,40^ On the other hand, budesonide is likely to have a favorable effect on the prevention of BPD when instilled endotracheally with surfactant, though more data on safety and effectiveness are necessary.^8,9,13,23,24,40^ When combined with a surfactant, budesonide is evenly distributed to the distal lungs with maximum efficacy and deposition,^38,41^ the role of budesonide in terms of promoting lung maturation is enhanced,^42^ and combined surfactant and budesonide treatment is associated with lower levels of interleukin in tracheal aspirates when compared to surfactant treatment alone.^9^ These positive effects suggest that endotracheal surfactant and budesonide combination therapy can be used in the treatment of NARDS, but randomized controlled studies with large patient groups are needed on this subject.

 Current studies also address new diagnostic, grading, and treatment options for NARDS, such as circular RNA expressions,^43^ lung-gut microbiota and lung-plasma tryptophan metabolites,^44^ lying position,^45^ and phospholipid-modified surfactant.^46^ Perhaps in the future, early recognition, accurate classification, and targeted treatments will be the definitive solution to NARDS. All patients included in our study were ventilated in the HFOV mode due to the development of respiratory failure in CMV, whereas it was previously reported that HFOV was not superior to CMV in reducing the incidence of mortality and BPD in moderate-severe perinatal-onset NARDS.^47^

 To the best of our knowledge, this is the first study evaluating the effects of endotracheal surfactant and budesonide combination therapy in a group of neonates with NARDS due to LONS, and the findings revealed an improvement in oxygenation status determined by OSI after treatment. The mortality rate in our study was higher than that reported in the literature (30% vs. 17%-24%), which may be because LONS was the cause of NARDS in all patients included in our study, and all patients were premature with other prematurity-related comorbidities. De Luca et al^2^ found that indirect NARDS was related to higher mortality compared to direct NARDS, and an infectious NARDS is associated with oxygen dependency and/or the need for respiratory support at 36 and 40 weeks postmenstrual age without influencing mortality.

 The main strength of our study was that the neonates were homogenous in terms of the etiology of NARDS, and the same treatment was applied to all of them by the same two investigators. In our study conducted in a perinatology center in a developing country with high birth rates, it was shown that endotracheal combined surfactant and budesonide treatment could improve ventilation in NARDS secondary to LONS without causing any undesirable side effects in the short term; however, it was impossible to determine whether the addition of budesonide to surfactant made an additional contribution. The most important limitation of the study was the lack of a control group, which made it impossible to judge the impact of the intervention. The other limitations were the cross-sectional retrospective design of the study, the small number of newborns included in the study, and the lack of long-term follow-ups. The final limitation was that we used OSI to define NARDS because we did not draw arterial blood, although the Montreux definition of NARDS includes the OI, and it is recommended not to use OSI.^1^

 It has been previously shown that the application of surfactant alone in the treatment of NARDS improves oxygenation,^29^ which conforms to our findings. Therefore, the post-treatment improvement in oxygenation observed in our patients may have been solely due to the effect of surfactant, rather than the addition of budesonide. As studies on NARDS-a syndrome defined only seven years ago continue to grow, the role of steroids in the treatment of NARDS will become clearer. Although conducting prospective, randomized controlled trials with a large patient population for this relatively rare syndrome is challenging, such studies are essential to establishing a definitive treatment approach.

## Conclusion

 In general, the decrease in oxygen demand and OSI along with an increase in SpO_2 _after treatment compared with pre-treatment values suggested that endotracheal surfactant and budesonide combination therapy could be used to improve oxygenation in NARDS secondary to LONS. Caution should be addressed considering the possibility that the undesirable effects of budesonide on BP fluctuations may lead to negative consequences. Prospective studies with large patient groups may help investigate the effect of endotracheal surfactant budesonide use in the treatment of NARDS with different etiologies on short- and long-term morbidities.

## References

[1] De Luca D, van Kaam AH, Tingay DG, Courtney SE, Danhaive O, Carnielli VP (2017). The Montreux definition of neonatal ARDS: biological and clinical background behind the description of a new entity. Lancet Respir Med.

[2] De Luca D, Tingay DG, van Kaam AH, Courtney SE, Kneyber MC, Tissieres P (2022). Epidemiology of Neonatal acute respiratory distress syndrome: prospective, multicenter, international cohort study. Pediatr Crit Care Med.

[3] Cave C, Samano D, Sharma AM, Dickinson J, Salomon J, Mahapatra S. Acute respiratory distress syndrome: a review of ARDS across the life course. J Investig Med. 2024:10815589241270612. 10.1177/10815589241270612. 39092841

[4] Shen L, Cai N, Wan S, Chen S (2023). Development and validation of a predictive model for early diagnosis of neonatal acute respiratory distress syndrome based on the Montreux definition. Front Pediatr.

[5] Amigoni A, Pettenazzo A, Stritoni V, Circelli M (2017). Surfactants in acute respiratory distress syndrome in infants and children: past, present and future. Clin Drug Investig.

[6] Emeriaud G, López-Fernández YM, Iyer NP, Bembea MM, Agulnik A, Barbaro RP (2023). Executive summary of the second international guidelines for the diagnosis and management of pediatric acute respiratory distress syndrome (PALICC-2). Pediatr Crit Care Med.

[7] De Luca D, Cogo P, Kneyber MC, Biban P, Semple MG, Perez-Gil J (2021). Surfactant therapies for pediatric and neonatal ARDS: ESPNIC expert consensus opinion for future research steps. Crit Care.

[8] Yeh TF, Lin HC, Chang CH, Wu TS, Su BH, Li TC (2008). Early intratracheal instillation of budesonide using surfactant as a vehicle to prevent chronic lung disease in preterm infants: a pilot study. Pediatrics.

[9] Yeh TF, Chen CM, Wu SY, Husan Z, Li TC, Hsieh WS (2016). Intratracheal administration of budesonide/surfactant to prevent bronchopulmonary dysplasia. Am J Respir Crit Care Med.

[10] Hillman NH, Kothe TB, Schmidt AF, Kemp MW, Royse E, Fee E (2020). Surfactant plus budesonide decreases lung and systemic responses to injurious ventilation in preterm sheep. Am J Physiol Lung Cell Mol Physiol.

[11] Baer B, McCaig L, Yamashita C, Veldhuizen R (2020). Exogenous surfactant as a pulmonary delivery vehicle for budesonide in vivo. Lung.

[12] Kothe TB, Sadiq FH, Burleyson N, Williams HL, Anderson C, Hillman NH (2020). Surfactant and budesonide for respiratory distress syndrome: an observational study. Pediatr Res.

[13] Venkataraman R, Kamaluddeen M, Hasan SU, Robertson HL, Lodha A (2017). Intratracheal administration of budesonide-surfactant in prevention of bronchopulmonary dysplasia in very low birth weight infants: a systematic review and meta-analysis. Pediatr Pulmonol.

[14] Kalola UK, Ambati S. Budesonide. In: StatPearls [Internet]. Treasure Island, FL: StatPearls Publishing; 2024. 33085348

[15] van de Loo M, van Kaam A, Offringa M, Doyle LW, Cooper C, Onland W (2024). Corticosteroids for the prevention and treatment of bronchopulmonary dysplasia: an overview of systematic reviews. Cochrane Database Syst Rev.

[16] Zhang M, Zhang W, Liao H (2024). Efficacy and safety of different inhaled corticosteroids for bronchopulmonary dysplasia prevention in preterm infants: a systematic review and meta-analysis. Respir Med Res.

[17] Anderson CD, Kothe TB, Josephsen JB, Sadiq FH, Burleyson N, Williams HL (2021). Budesonide mixed with surfactant did not affect neurodevelopmental outcomes at 6 or 18-months corrected age in observational cohorts. J Perinatol.

[18] Kuo HT, Lin HC, Tsai CH, Chouc IC, Yeh TF (2010). A follow-up study of preterm infants given budesonide using surfactant as a vehicle to prevent chronic lung disease in preterm infants. J Pediatr.

[19] Nobile S, Di Sipio Morgia C, Hall M (2024). Long-term effects of intratracheal budesonide and surfactant for the prevention of bronchopulmonary dysplasia: a narrative review. Am J Perinatol.

[20] Moschino L, Nardo D, Bonadies L, Stocchero M, Res G, Priante E (2021). Intra-tracheal surfactant/budesonide versus surfactant alone: comparison of two consecutive cohorts of extremely preterm infants. Pediatr Pulmonol.

[21] Tang W, Chen S, Shi D, Ai T, Zhang L, Huang Y (2022). Effectiveness and safety of early combined utilization of budesonide and surfactant by airway for bronchopulmonary dysplasia prevention in premature infants with RDS: a meta-analysis. Pediatr Pulmonol.

[22] Szabó H, Baraldi E, Colin AA (2022). Corticosteroids in the prevention and treatment of infants with bronchopulmonary dysplasia: part II Inhaled corticosteroids alone or in combination with surfactants. Pediatr Pulmonol.

[23] Dolma K, Zayek M, Gurung A, Eyal F (2024). Intratracheal instillation of budesonide-surfactant for prevention of bronchopulmonary dysplasia in extremely premature infants. Am J Perinatol.

[24] Manley BJ, Kamlin COF, Donath S, Huang L, Birch P, Cheong JLY (2023). Intratracheal budesonide mixed with surfactant to increase survival free of bronchopulmonary dysplasia in extremely preterm infants: study protocol for the international, multicenter, randomized PLUSS trial. Trials.

[25] Phattraprayoon N, Ungtrakul T, Tangamornsuksan W (2021). The effects of different types of steroids on clinical outcomes in neonates with meconium aspiration syndrome: a systematic review, meta-analysis and GRADE assessment. Medicina (Kaunas).

[26] Bruschettini M, Hassan KO, Romantsik O, Banzi R, Calevo MG, Moresco L (2022). Interventions for the management of transient tachypnoea of the newborn - an overview of systematic reviews. Cochrane Database Syst Rev.

[27] Hu Y, Chen X, Wang F, Li C, Yue W, Wei H (2024). Risk factors of neonatal acute respiratory distress syndrome based on the Montreux definition in neonates with sepsis: a retrospective case-control study. Am J Perinatol.

[28] Lim AM, Lee JH, Quek BH. Epidemiology of neonatal acute respiratory distress syndrome in a neonatal ICU: a retrospective study utilising the Montreux definition. Singapore Med J. 2023. 10.4103/singaporemedj.SMJ-2022-156. PMC13143323 37870039

[29] Rong Z, Mo L, Pan R, Zhu X, Cheng H, Li M (2021). Bovine surfactant in the treatment of pneumonia-induced-neonatal acute respiratory distress syndrome (NARDS) in neonates beyond 34 weeks of gestation: a multicentre, randomized, assessor-blinded, placebo-controlled trial. Eur J Pediatr.

[30] Chen L, Li J, Shi Y (2023). Clinical characteristics and outcomes in neonates with perinatal acute respiratory distress syndrome in China: a national, multicentre, cross-sectional study. EClinicalMedicine.

[31] Deliloglu B, Tuzun F, Cengiz MM, Ozkan H, Duman N (2020). Endotracheal surfactant combined with budesonide for neonatal ARDS. Front Pediatr.

[32] Satar M, Arısoy AE, Çelik İ H (2018). Turkish Neonatal Society guideline on neonatal infections-diagnosis and treatment. Turk Pediatri Ars.

[33] Miall L, Wallis S (2011). The management of respiratory distress in the moderately preterm newborn infant. Arch Dis Child Educ Pract Ed.

[34] Muniraman HK, Song AY, Ramanathan R, Fletcher KL, Kibe R, Ding L (2019). Evaluation of oxygen saturation index compared with oxygenation index in neonates with hypoxemic respiratory failure. JAMA Netw Open.

[35] Ricci F, Catozzi C, Ravanetti F, Murgia X, D’Aló F, Macchidani N (2017). In vitro and in vivo characterization of poractant alfa supplemented with budesonide for safe and effective intratracheal administration. Pediatr Res.

[36] Chen CM, Chang CH, Chao CH, Wang MH, Yeh TF (2019). Biophysical and chemical stability of surfactant/budesonide and the pulmonary distribution following intra-tracheal administration. Drug Deliv.

[37] Zubrow AB, Hulman S, Kushner H, Falkner B (1995). Determinants of blood pressure in infants admitted to neonatal intensive care units: a prospective multicenter study Philadelphia Neonatal Blood Pressure Study Group. J Perinatol.

[38] Boel L, Banerjee S, Chakraborty M (2018). Postnatal steroids in extreme preterm infants: intra-tracheal instillation using surfactant as a vehicle. Paediatr Respir Rev.

[39] Shah SS, Ohlsson A, Halliday HL, Shah VS (2017). Inhaled versus systemic corticosteroids for the treatment of bronchopulmonary dysplasia in ventilated very low birth weight preterm infants. Cochrane Database Syst Rev.

[40] Perrone S, Orlando S, Petrolini C, Marinelli F, Moretti S, Corradi M (2023). Current concepts of corticosteroids use for the prevention of bronchopulmonary dysplasia. Curr Pediatr Rev.

[41] Zecchi R, Franceschi P, Tigli L, Pioselli B, Mileo V, Murgia X (2021). Surfactant-assisted distal pulmonary distribution of budesonide revealed by mass spectrometry imaging. Pharmaceutics.

[42] Li L, Yang C, Feng X, Du Y, Zhang Z, Zhang Y (2018). Effects of intratracheal budesonide during early postnatal life on lung maturity of premature fetal rabbits. Pediatr Pulmonol.

[43] Zhou H, Chanda B, Chen YF, Wang XJ, You MY, Zhang YH (2021). Microarray and bioinformatics analysis of circular RNA differential expression in newborns with acute respiratory distress syndrome. Front Pediatr.

[44] Yang J, He Y, Ai Q, Liu C, Ruan Q, Shi Y (2024). Lung-gut microbiota and tryptophan metabolites changes in neonatal acute respiratory distress syndrome. J Inflamm Res.

[45] Loi B, Regiroli G, Foligno S, Centorrino R, Yousef N, Vedovelli L (2023). Respiratory and haemodynamic effects of 6h-pronation in neonates recovering from respiratory distress syndrome, or affected by acute respiratory distress syndrome or evolving bronchopulmonary dysplasia: a prospective, physiological, crossover, controlled cohort study. EClinicalMedicine.

[46] Kupsch S, Eggers LF, Spengler D, Gisch N, Goldmann T, Fehrenbach H (2022). Characterization of phospholipid-modified lung surfactant in vitro and in a neonatal ARDS model reveals anti-inflammatory potential and surfactant lipidome signatures. Eur J Pharm Sci.

[47] Liu K, Chen L, Xiong J, Xie S, Hu Y, Shi Y (2021). HFOV vs CMV for neonates with moderate-to-severe perinatal onset acute respiratory distress syndrome (NARDS): a propensity score analysis. Eur J Pediatr.

